# A homozygous *EVC* mutation in a prenatal fetus with Ellis–van Creveld syndrome

**DOI:** 10.1002/mgg3.2183

**Published:** 2023-05-09

**Authors:** Jie Wang, Xiaohua Wang, Yueqi Jia, Xiangnan Li, Guohui Liu, Rula Sa, Haiquan Yu

**Affiliations:** ^1^ State Key Laboratory of Reproductive Regulation and Breeding of Grassland Livestock (RRBGL) Inner Mongolia University Hohhot 010070 China; ^2^ Department of Genetics Inner Mongolia Maternity and Child Health Care Hospital Hohhot 010010 China; ^3^ Department of Ultrasonic Medicine Inner Mongolia Maternity and Child Health Care Hospital Hohhot 010010 China

**Keywords:** aberrant splicing effect, Ellis–van Creveld syndrome, *EVC*, skeletal dysplasia

## Abstract

**Background:**

Ellis–van Creveld (EvC) syndrome, caused by variants in *EVC*, is a rare genetic skeletal dysplasia. Its clinical phenotype is highly diverse. EvC syndrome is rarely reported in prenatal stages because its presentation overlaps with other diseases.

**Methods:**

A Chinese pedigree diagnosed with EvC syndrome was enrolled in this study. Whole‐exome sequencing (WES) was applied in the proband to screen potential genetic variant(s), and then Sanger sequencing was used to identify the variant in family members. Minigene experiments were applied.

**Results:**

WES identified a homozygous variant (NM_153717.3:c.153_174 + 42del) in *EVC* which was inherited from the heterozygous parents and confirmed by Sanger sequencing. Further experiments demonstrated that this variant disrupts the canonical splicing site and produces a new splicing site at NM_153717.3: c.‐164_174del, which ultimately leads to a 337 bp deletion at the 3′ end of exon 1 and loss of the start codon.

**Conclusion:**

This is the first reported case of EvC syndrome based on a splicing variant and detailed delineation of the aberrant splicing effect in the fetus. Our study demonstrates the pathogenesis of this new variant, expands the spectrum of *EVC* mutations, and demonstrates that WES is a powerful tool in the clinical diagnosis of diseases with genetic heterogeneity.

## Graphical abstract

Ellis–van Creveld (EvC) syndrome, caused by variants in the EVC gene, is a rare genetic skeletal dysplasia. WES identified a homozygous variant (NM_153717.3:c.153_174 + 42del) in a Chinese pedigree diagnosed with EvC syndrome. Further experiments demonstrated that this novel variant disrupts the canonical splicing site and produces a new splicing site at NM_153717.3: c.‐164_174del, which ultimately leads to a 337 bp deletion at the 3′ end of exon 1 and loss of the start codon.

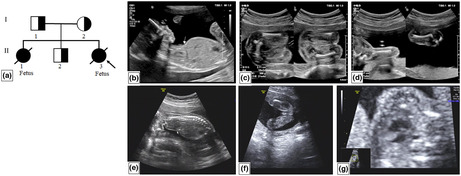

## 1 INTRODUCTION

Skeletal dysplasia is a broad spectrum of syndromes with skeletal developmental abnormalities and considerable heterogeneity in the background loci and alleles. Fetal skeletal structure malformations are prevalent in approximately 3% of pregnancies and account for nearly 20% of prenatal deaths (Osterman et al., 2015; Persson et al., 2017).

Ellis–van Creveld Syndrome (EvC syndrome, OMIM:225500), also called “chondroectodermal dysplasia” or “mesoectodermal dysplasia,” is a rare genetic skeletal disorder. EvC syndrome is inherited in an autosomal recessive pattern, with an estimated prevalence of 7 per 1,000,000 in the general population (Stoll et al., 1989). However, the incidence is as high as 5 per 1000 live births in the Old Order Amish population of Lancaster Country, Pennsylvania, USA (Kamal et al., 2013). EvC syndrome is characterized by chondrodystrophy, ectodermal dysplasia, and congenital heart defects (Al‐Fardan & Al‐Qattan, 2017). In the prenatal stage, it is characterized by a narrow thorax, obvious shortening of the long bones, postaxial polydactyly, and cardiac defects (Guschmann et al., 1999; Horigome et al., 1997). In clinical evaluation, EvC syndrome is likely to be misdiagnosed as Smith–Lemli–Opitz syndrome (OMIM 270400) or Hydrolethalus syndrome (OMIM 236680) because they share similar phenotypes, including cardiac and skeletal defects (Parilla et al., 2003; Schramm et al., 2009; Witters et al., 2008).

Approximate 70% of EvC syndrome cases are attributed to disease‐causing variants in two genes, *EVC* (OMIM 604831) and *EVC2* (OMIM 607261), while a very small proportion have been linked with other genes (D'Asdia et al., 2013; Ruiz‐Perez & Goodship, 2009; Tompson et al., 2007). *EVC* and *EVC2* are located on chromosome 4p16, separated by 2.6 Kb of genomic sequence (Galdzicka et al., 2002; Ruiz‐Perez et al., 2000). Both genes encode single‐pass type I transmembrane proteins expressed in the heart, kidney, lungs, and developing bones (Aziz et al., 2016). These proteins transduce extracellular signals to the nucleus via the hedgehog signaling pathway (Caparrós‐Martín et al., 2013; Pusapati et al., 2014). Patients with EvC syndrome more often have a variant in *EVC* than in *EVC2* (D'Asdia et al., 2013). To date, the number of ClinVar pathogenic and likely pathogenic variants in *EVC* and *EVC2* is 105 and 124, respectively (202203 version). The variant consequences include frameshift, nonsense, splicing, start codon loss, and missense.

Whole‐exome sequencing (WES) has revolutionized clinical diagnosis of monogenic disorders in recent years. It is a powerful tool that enables clinicians to achieve an accurate diagnosis of diseases with variable and complex phenotypes (Xue et al., 2015). Recent studies showed that the diagnostic yield of WES was 15%–24% in fetal skeletal abnormalities (Lord et al., 2019; Petrovski et al., 2019). Approximately 85% of the variants in diagnosed cases were found in the coding region or at canonical splice sites (Choi et al., 2009). Disease‐causing variants in introns have been reported less often because of challenges in interpreting their clinical significance, which requires splicing or functional experiments.

In this study, we report a fetus diagnosed with EvC syndrome with the aid of WES. The patient presented fetal cardiac and skeletal dysplasia symptoms. WES identified a homozygous variant (NM_153717.3:c.153_174 + 42del) in *EVC* which was inherited from the heterozygous parents, as confirmed by Sanger sequencing. Further experiments demonstrated that this variant disrupts the canonical splice site and creates a new 5′ splice site in exon 1, resulting in a 337 bp deletion at the 3′ end of exon 1 and loss of the start codon. Our study demonstrates the pathogenesis of this new variant, expands the spectrum of *EVC* mutations, and demonstrates that WES is a powerful tool in the clinical diagnosis of diseases with genetic heterogeneity.

## 2 MATERIALS AND METHODS

### 2.1 Ethical compliance

The study was approved from the Ethics Review Committee of Inner Mongolia Maternity and Child Health Care Hospital (approval number: 2020‐073). The parent provided written consent on behalf of the child and fetus participant. The written consent was also received from the patient's parents for their own participation in the study.

### 2.2 Patient and samples collection

A 34‐year‐old pregnant woman was evaluated due to ultrasound abnormalities. To detect the disease‐causing variant, samples were collected for genetic analysis (with the agreement of the family) from the product of the third conception, the blood of parents, and the health boy. Genomic DNA was extracted from tissues or blood leukocytes using a QIAamp DNA Mini Kit (Qiagen) according to the manufacturer's instructions. DNA quality was checked with a NanoDrop 8000 spectrophotometer (Thermo Fisher Scientific).

### 2.3 Genetic analysis

A single nucleotide polymorphism (SNP) array analysis was performed on the proband only. Analysis of SNP‐array data was performed with the Chromosome Analysis Suite software, version 4.0 (Affymetrix). No chromosomal abnormalities were identified.

Exome capture was done using Roche KAPA HyperExome probes according to the manufacturer's instructions (Roche Diagnostics). The library was constructed and sequenced at paired‐end 100 (PE 100) on an MGISEQ‐2000 sequencer (BGI Genomics).

The sequencing data were aligned to the human reference genome (hg19/GRCh37) using BWA (Li & Durbin, 2009). GATK (DePristo et al., 2011) and VEP (McLaren et al., 2016) were employed for variant calling and variant annotation. The allele frequency of each variant was aggregated from the gnomAD database (Karczewski et al., 2020). Common SNPs, defined as a minor allele frequency >0.1%, were filtered. SIFT (Ng & Henikoff, 2003), PolyPhen‐2 (Adzhubei et al., 2013), and MutationTaster (Schwarz et al., 2010) were used to predict the pathogenicity of missense variants, while SpliceAI (Jaganathan et al., 2019), MaxEntScan (Yeo & Burge, 2004), and Human Splicing Finder (Desmet et al., 2009) were used to evaluate the effects of splicing variants. Finally, variants were interpreted based on the variant interpretation guidelines recommended by the American College of Medical Genetics and Genomics and the Association for Molecular Pathology (ACMG/AMP) (Richards et al., 2015). The final classifications were grouped into five tiers: pathogenic, likely pathogenic, uncertain significance, likely benign or benign (Richards et al., 2015).

### 2.4 Minigene construction

We cloned the entire sequence of exon 1 (354 bp) and partial surrounding intron1 (1773 bp) and partial exon2 (126 bp) of wild‐type and mutant *EVC* into pcDNA3.1 to construct wild‐type and mutant pcDNA3.1‐EVC, respectively. The mutant *EVC* (c.153_174 + 42del) was obtained by nested PCR using genomic DNA from the proband's father as a template. HeLa and HEK293T cells were purchased from the China Center for Type Culture Collection. The cells were cultured on high‐glucose Dulbecco's Modified Eagle Medium (DMEM; Gibco) containing 10% fetal bovine serum (Gibco) and 1% Penicillin–Streptomycin (Gibco) at a constant temperature of 37°C in incubators with 5% CO_2_ and saturated humidity.

### 2.5 Cell transient transfection

Constructs containing the wild‐type and mutant EVC transcripts were transfected into HeLa and HEK293T cells using Lipofectamine 2000 (Yeasen Biotech). Total RNA was extracted from HeLa and HEK293T cells at 48 h post‐transfection using Trizol Reagent (Takara), and the cDNA was transcribed using a reverse transcription kit (Yeasen Biotech). Minigene‐specific cDNA was amplified using plasmid‐specific primers. Splicing pattern analysis was done via electrophoresis on 2% agarose gels and with Sanger sequencing. The primers for *EVC* gene amplification are listed in Table S1.

## 3 RESULTS

### 3.1 Clinical findings

In October 2021, a 34‐year‐old pregnant woman (G3P1) was admitted to our clinic (Inner Mongolia Maternity and Child Health Care Hospital, China) for counseling. Her husband was also 34 years old, and both are Chinese Han.

**FIGURE 1:**
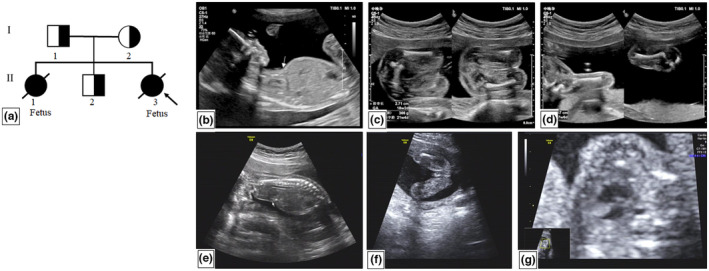
The pedigree and the prenatal transabdominal ultrasound. (a) the pedigree; (b) thoracic narrowness (II‐3); (c) short femur (II‐3); (d) short humerus (II‐3); (e) thoracic narrowness (II‐1); (f) short femur (II‐1); (g) single atrium and single ventricle (II‐1). White arrows represent thoracoabdominal asynchrony.

The couple had had two previous conceptions. The pregnancy in 2014 was terminated at 23^+4^ weeks of gestation due to fetal abnormalities, including short humerus length (<3 standard deviation, SD), short femur length (<3 SD), thoracic dysplasia, aortic stenosis, a single atrium, and a single ventricle at 22^+5^ weeks of gestation (Figure 1e–g). No other family history was reported. Consanguineous marriage was denied by the family.

In 2016, the woman delivered a healthy boy by natural labor at full term (Figure 1 II‐2). No obvious symptoms were observed at the follow‐up (6 years of age).

In 2021, the couple had a third conception. Combined screening at the first trimester showed a low risk for trisomy 13, 18, and 21. At 14^+3^ weeks of gestation, the ultrasound scans were normal. However, the humerus length and femur length were short (<3 SD) at 18^+5^ weeks of gestation. Multiple abnormalities were observed at 21^+4^ weeks, including short humerus length (<3 SD), short femur length (<3 SD), postaxial polydactyly, thoracic dysplasia, and a complete atrioventricular septal defect (Figure 1c,d). The pregnancy was subsequently terminated at 23^+1^ weeks. Fetal anomalies were obvious, including six fingers on both hands, and short arms and legs.

### 3.2 Genetic analysis

**FIGURE 2:**
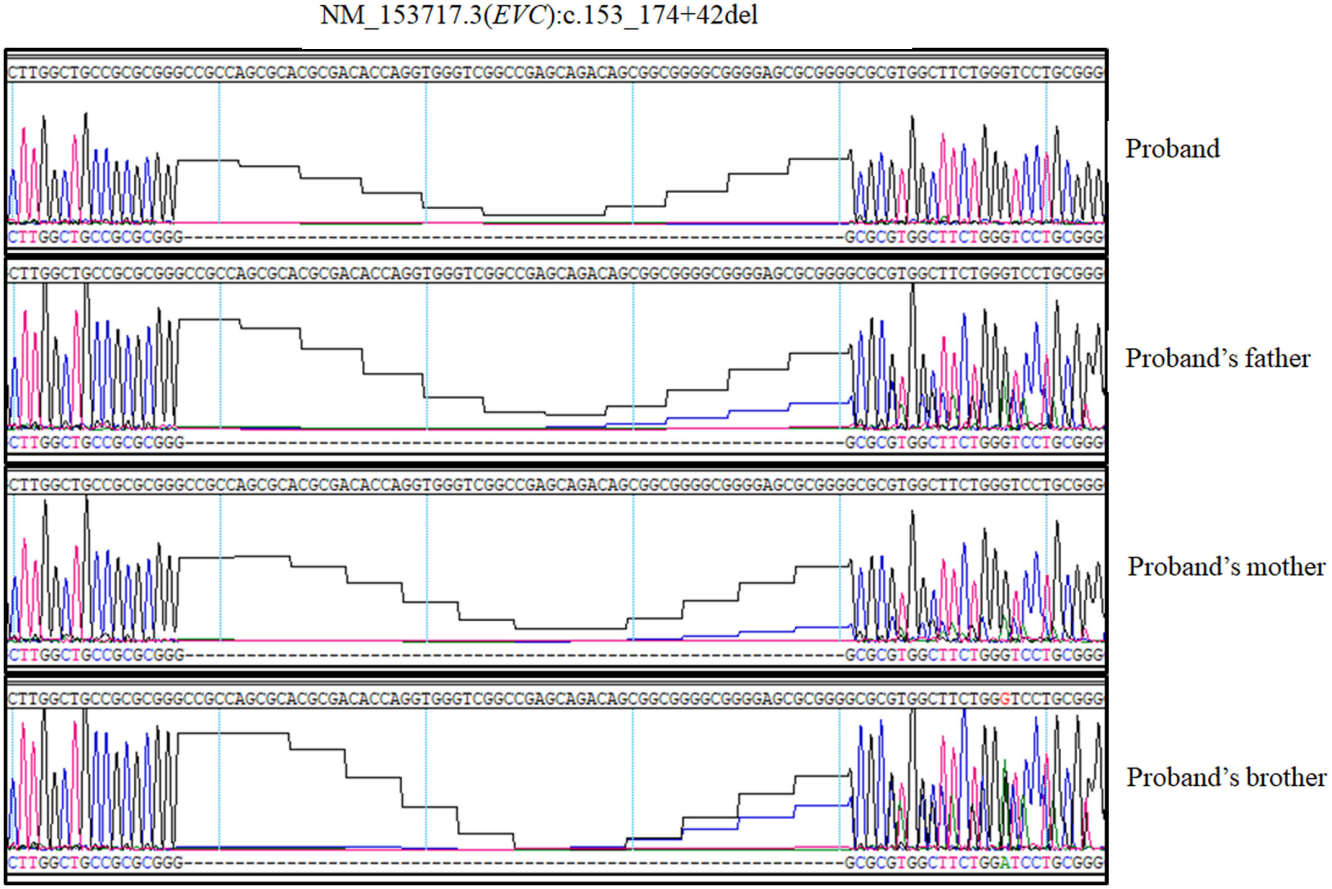
Sanger sequencing confirmed the zygosity of NM_153717.3(EVC):c.153_174 + 42del in the pedigree. The proband (II‐3) is in homozygous and the parents and brother are in heterozygous.

WES was carried out for the proband fetus (Figure 1, II‐3) and her parents (Figure 1, I‐1, I‐2) in parallel. A mean coverage at least 20× was achieved in the WES data of the proband, the proband's father, and the proband's mother for 99.81%, 99.97%, and 99.82% of the targeted regions, respectively. WES data revealed that the pregnant woman and her husband did not have a close genetic relationship. Sequencing variants were prioritized based on allele frequency, segregation analysis, and in silico algorithmic analysis.

Interestingly, a homozygous variant (NM_153717.3:c.153_174 + 42del) in *EVC* was identified. It was homozygous in the proband and heterozygous in the parents. The healthy boy (II‐2) was also heterozygous (Figure 2). The variant was confirmed in all samples by Sanger sequencing (Figure 2). The primer sequences are listed in Table S2. The NM_153717.3:c.153_174 + 42del variant did not exist in the gnomAD database (Karczewski et al., 2020). It was predicted to result in a frameshift, p.(Arg52Lysfs*57) and classified as “likely pathogenic” based on ACMG/AMP criteria: PVS1 and PM2. After adding the functional study (PS3) carried in this study, NM_153717.3:c.153_174+42del was classified as “pathogenic.”

### 3.3 In vitro splicing assay

Since NM_153717.3:c.153_174+42del was predicted to disturb splicing based on computational analysis with SpliceAI (Jaganathan et al., 2019), MaxEntScan (Yeo & Burge, 2004), and Human Splicing Finder (Desmet et al., 2009), we constructed a recombined plasmid, pcDNA3.1‐EVC, for a minigene splicing assay to investigate the impact of the variant.

**FIGURE 3:**
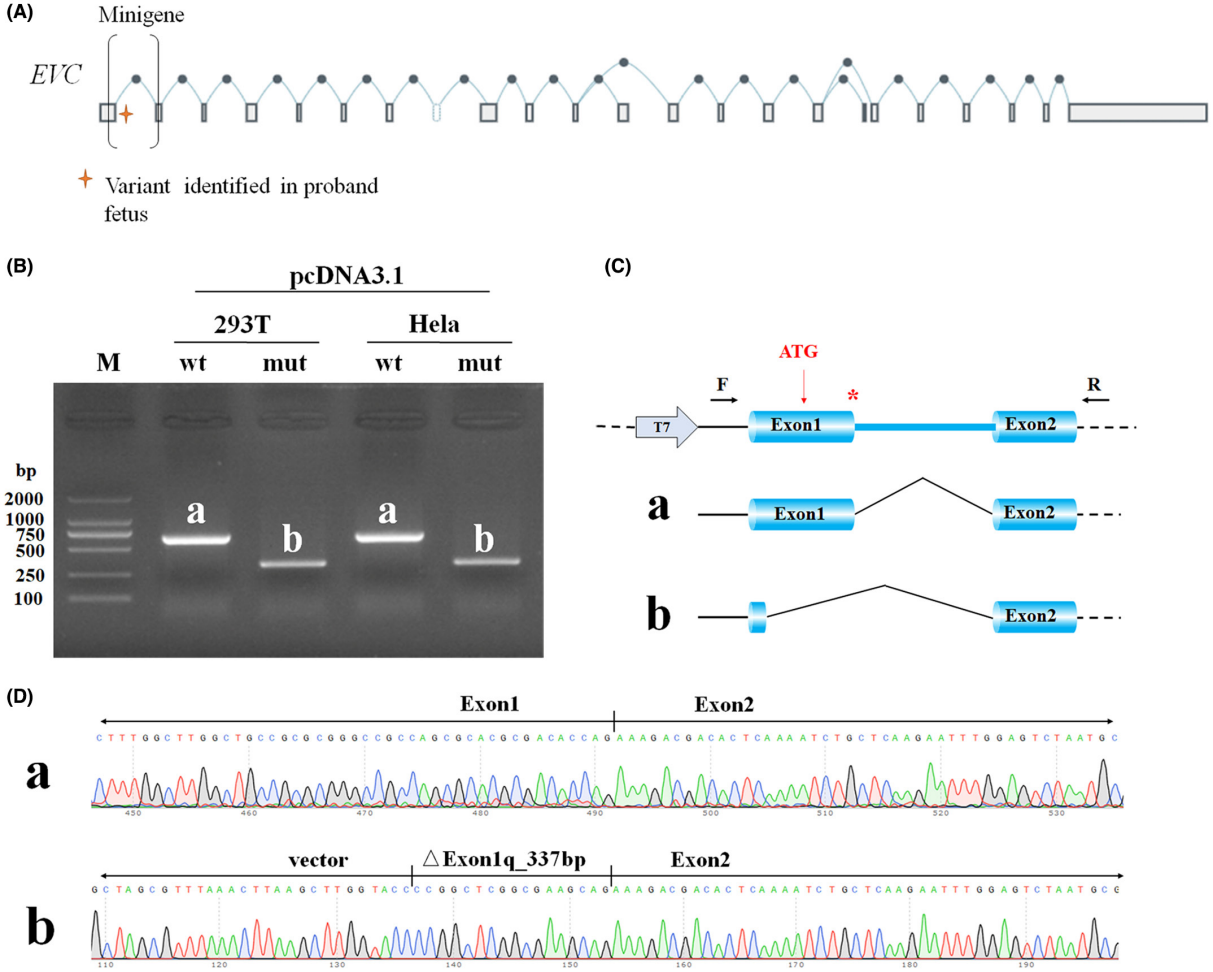
The minigene splicing assay for NM_153717.3(EVC):c.153_174 + 42del in HEK293T and Hela cells with pcDNA3.1 vector. A. The schematic representation of splicing junctions of EVC. B. Agarose gel electrophoresis of RT‐PCR products in HEK293T and Hela‐7 cells showed a shorter fragment in variant group compared to wild type. C. The Schematic diagram showed the splicing events of wild type (a) and NM_153717.3(EVC):c.153_174+42del (b) in the minigene experiments. The arrows indicate the primers used for RT‐PCR. D. The Sanger sequencing of RT‐PCR product confirmed 337 base pair deletion of exon 1 at the 3′ end in the variant group (b) compared to the wild type (a).

**FIGURE 4:**
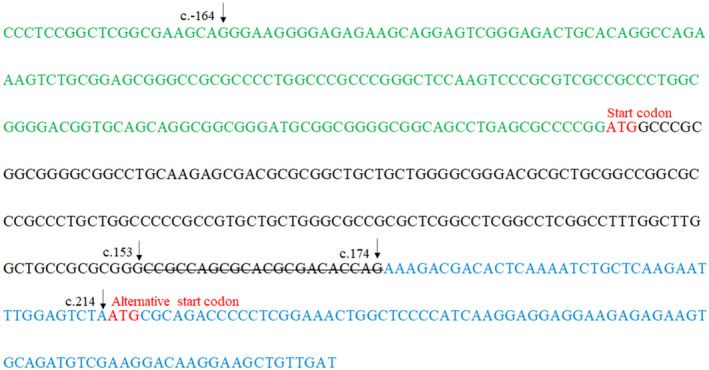
A schematic diagram of exon 1 and exon 2 in the EVC gene. The untranslated sequence of exon 1 is in green. The coding sequence of exon 1 is in black. The coding sequence of exon 2 is in blue. The start codon is in red.

The minigene splicing assay demonstrated that the mutant construct produced shorter fragments in both HeLa and HEK293T cells (Figure 3b), indicating an aberrant splicing effect (Figure 3c). Sanger sequencing showed that the mutation creates a new splicing site at c.‐164_174del, resulting in a 337 bp deletion at the 3′ end of exon 1 (Figure 3d). Interestingly, the start codon was inside the 337 bp deletion sequence (Figures 3c and 4). The protein would therefore translate from the nearest alternative start codon at c.215 (Figure 4). That is, the new start codon is out of phase so that the mutant transcript will have no similarity with EVC. The out of frame transcription may lead to nonsense‐mediated mRNA decay (Lewis et al., 2003).

## 4 DISCUSSION

Here, we report clinical and genetic data from a proband with a non‐consanguineous pedigree suffering from EvC syndrome. An earlier conception by the same couple had similar ultrasound phenotypes to the proband, including short humerus length, short femur length, thoracic dysplasia, and aortic stenosis. However, only the proband had postaxial polydactyly. WES of the proband identified a variant (NM_153717.3:c.153_174+42del) in *EVC*. Segregation analysis confirmed this as the disease‐causing variant. A minigene splicing assay demonstrated that the variant disrupts the canonical splicing site, producing a new splicing site in the untranslated region (NM_153717.3:c.‐164_174del) which leads to a 337 bp deletion at the 3′ end of exon 1 and loss of the start codon. To our knowledge, this is the first study reporting a deletion variant in a clinical prenatal case supplemented with a splicing assay.

EvC syndrome is characterized by genetic heterogeneity (in terms of alleles and loci). Allelic heterogeneity leads to heterogeneity in the clinical phenotype. For example, a number of variants (such as c.1868T>C and c.2653C>T) cause a mild phenotype (Shen et al., 2011; Ulucan et al., 2008). In our study, the two fetuses displayed differences in postaxial polydactyly. The variable phenotype poses challenges for clinicians in achieving an accurate clinical diagnosis.

Locus heterogeneity presents a different challenge. Pathogenic variants in two genes (*EVC* and *EVC2*) can cause EvC syndrome. *EVC* is on chromosome 4p16.2, contains 21 exons, and encodes a 992‐amino acid protein. It orients head‐to‐head with its homolog, *EVC2*, which also encodes a single‐pass type I transmembrane protein (Al‐Fardan & Al‐Qattan, 2017). These genes are specifically expressed in the developing skeleton, heart, kidneys, and lungs (Aziz et al., 2016). The two proteins mutually interact to form a complex in the primary cilium membrane (EvC zone) which is essential for endochondral growth and intramembranous ossification (Dorn et al., 2012). Since the two genes are in the same pathway and interact each other (Caparrós‐Martín et al., 2013; Pusapati et al., 2014), it is extremely hard to distinguish them based solely on phenotype.

ClinVar had 105 submitted pathogenic and likely pathogenic variants in *EVC*. Most of them (*n* = 98, 93%) are null variants, including frameshift, nonsense, canonical splicing, and start codon loss, which is consistent with a loss of function (Ruiz‐Perez et al., 2007). Deletion variants were very rare (*n* = 6). Although NM_153717.3:c.153_174+42del had a single record in ClinVar, classified as “likely pathogenic,” it was supported neither by splicing experiments nor in the public literature. An effective assay to evaluate the splicing effect is of importance to understand the pathogenesis. Our study provides the first clinical case and solid segregation evidence. In addition, the minigene splicing assay confirmed an aberrant splicing event and start codon loss, which ultimately leads to nonsense‐mediated mRNA decay (Lewis et al., 2003). In light of these findings, the variant was re‐classified as “pathogenic” based on the ACMG/AMP variant interpretation guidelines (Richards et al., 2015).

One caveat in this study is that although the couple denied consanguineous marriage, they come from a remote rural population, so a distant genetic relationship cannot be ruled out. Further analysis of WES data confirmed that they do not have a close genetic relationship. It is possible that NM_153717.3:c.153_174+42del in *EVC* derives from a founder effect. Population studies are warranted in the future.

In conclusion, we report a deletion variant supported by a minigene splicing assay in a fetus. The variant disrupts the canonical splicing site, producing a new splicing site in the untranslated region (NM_153717.3:c.‐164_174del) and leading to a 337 bp deletion at the 3′ end of exon 1 and loss of the start codon. Our study demonstrates the pathogenesis of this new variant and expands the spectrum of reported mutations in *EVC*. Furthermore, these findings demonstrate that WES is a powerful tool in the clinical diagnosis of diseases with genetic heterogeneity.

## AUTHOR CONTRIBUTIONS

J.W. and H.Y. conceived and designed the study, coordinated the research, and wrote the manuscript. X.W. and Y.J. designed and commented on the manuscript draft. J.W., X.L., G.L., and R.S. coordinated collection of samples, undertook genetic counseling, and analyzed those data. J.W. and H.Y. reviewed and revised the manuscript. All authors read and approved the final manuscript.

## FUNDING INFORMATION

This work was supported by the Key Technology Research Plan Project of Inner Mongolia Autonomous Region (2021GG0153 to H.Y.), the Key Technology Research Plan Project of Inner Mongolia Autonomous Region(2021GG0130 to X.W.), and the Healthcare Science and Technology Plan Project of Inner Mongolia Health Commission (202201138). The funds were mainly used for minigene experiments.

## CONFLICT OF INTEREST STATEMENT

The authors declare that they have no competing interests.

## Supporting information


Table S1.
Table S2.
Table S3.

## Data Availability

The data generated for this study are included in the supplementary materials. And the WES data are not publicly available due to privacy or ethical restrictions.
