# Supplementary material for: A homozygous EVC mutation in a prenatal fetus with Ellis–van Creveld syndrome
Source: Mol Genet Genomic Med. 2023 May 9;11(8):e2183. doi: 10.1002/mgg3.2183 (PMC10422067; doi:10.1002/mgg3.2183)
Supplement: Supplementary file 1 — Table S1. Table S2. Table S3. [file MGG3-11-e2183-s001.docx]

**Supplementary Table 1. The primers used in minigene assay**

| Primer | Primer sequences (5’-3’) |
| --- | --- |
| EVC-487- F | ctctctacccatcgtctcag |
| EVC-727- F | gcgtcttcatcaggaggatg |
| EVC-3170- R | aggagtcactggaggtgttt |
| EVC-3519- R | gttggtcaggctggtctcta |
| EVC-8257- F | tctgcactgtgtttggtggt |
| EVC-8518- F | gcaagtacaggaattggcaa |
| EVC-9404- R | gaaaacattctcaggggggt |
| EVC-9651- R | ggcacatccacacagttgact |
| DNA3.1-EVC-KpnI-F | GCTTGGTACCCCGGCTCGGCGAAGCAGGGA |
| DNA3.1-EVC-F | gaggtggtggttagtggcatcctggaggcc |
| DNA3.1-EVC-R | ggcctccaggatgccactaaccaccacctc |
| DNA3.1-EVC-EcoRI-R | TGCAGAATTCATCAACAGCTTCCTTGTCCT |

**Supplementary Table 2. The primers used in Sanger sequencing**

| Primer | Primer sequences (5’-3’) | Length |
| --- | --- | --- |
| EVC- F | GGGGTGGCTGAAAGTTTTGAGC | 508bp |
| EVC- R | AAGTTCCCAACCAGGCTCAAGG |  |

**Supplementary Table 3. The ClinVar variants presumed to affect splicing events in the *EVC* gene (NM_153717.3)**

| # | cHGVS | ClinVar  ID | Consequence | EXON/Intron | ClinVar pathogenecity | Experiment investigating splicing event | Clinical case reported in public literature | Patient type |
| --- | --- | --- | --- | --- | --- | --- | --- | --- |
| 1 | c.801+2T>G | 557972 | splice-5 | IVS6 | LP | No | No | - |
| 2 | c.939+1G>C | 554221 | splice-5 | IVS7 | LP | No | No | - |
| 3 | c.2782+1del | 557670 | splice-5 | EX19 | P/LP | No | No | - |
| 4 | c.2304+2T>C | 552015 | splice-5 | IVS15 | P/LP | No | No | - |
| 5 | c.2449+1G>A | 551324 | splice-5 | IVS16 | LP | No | No | - |
| 6 | c.2782+1G>T | 556536 | splice-5 | IVS19 | P/LP | No | No | - |
| 7 | c.2097+1G>T | 556814 | splice-5 | IVS14 | LP | No | No | - |
| 8 | c.702+1G>A | 553747 | splice-5 | IVS5 | LP | No | No | - |
| 9 | c.2561+1G>A | 551032 | splice-5 | IVS17 | LP | No | No | - |
| 10 | c.2688+1G>C | 554228 | splice-5 | IVS18 | LP | No | No | - |
| 11 | c.1886+1G>C | 861541 | splice-5 | IVS13 | LP | No | No | - |
| 12 | c.617+1G>A | 845441 | splice-5 | IVS4 | LP | No | No | - |
| 13 | c.1886+2T>G | 941560 | splice-5 | IVS13 | LP | No | No | - |
| 14 | c.939+1G>A | 951386 | splice-5 | IVS7 | LP | No | No | - |
| 15 | c.2561+1dup | 1068990 | splice-5 | EX17 | P | No | No | - |
| 16 | c.802-1G>C | 371398 | splice-3 | IVS6 | LP | No | No | - |
| 17 | c.703-1G>A | 553461 | splice-3 | IVS5 | LP | No | YES, PMID17024374 | Unknown |
| 18 | c.1777-2A>G | 551792 | splice-3 | IVS12 | P/LP | No | YES, PMID17024374 | Unknown |
| 19 | c.1777-1G>A | 554484 | splice-3 | IVS12 | LP | No | YES, PMID23220543 | Fetus |
| 20 | c.2098-1G>A | 554844 | splice-3 | IVS14 | LP | No | No | - |
| 21 | c.301-1G>A | 556523 | splice-3 | IVS2 | LP | No | No | - |
| 22 | c.1564-2A>G | 557788 | splice-3 | IVS11 | LP | No | No | - |
| 23 | c.2562-2A>G | 646416 | splice-3 | IVS17 | LP | No | No | - |
| 24 | c.2450-1G>A | 917938 | splice-3 | IVS16 | LP | No | No | - |
| 25 | c.703-1G>C | 944207 | splice-3 | IVS5 | LP | No | YES, PMID17024374 | Unknown |
| 26 | c.2098-2A>G | 951645 | splice-3 | IVS14 | LP | No | No | - |
| 27 | c.1465-1G>A | 998010 | splice-3 | IVS10 | LP | YES, PMID26621368, minigene assay | YES, PMID26621368 | Child, 1.5 years |
| 28 | c.175-2A>G | 1066868 | splice-3 | IVS1 | LP | No | YES, PMID19810119 | Unknown |
| 29 | c.2098-1G>C | 1210402 | splice-3 | IVS14 | LP | No | No | - |
| 30 | c.1886+5G>T | 5338 | splice_region | IVS13 | P | YES, PMID17024374, An RT-PCR performed on lymphoblast cell lines) | YES, PMID17024374 | Unknown |
| 31 | c.2894+3A>G | 488510 | splice_region | IVS20 | P/LP | No | YES, PMID23220543  PMID29321360 | Infant and Child |
| 32 | c.175-9G>A | 558765 | splice_region | IVS1 | LP | No | No | - |
| 33 | c.1465-3_1472del | 557196 | IVS | IVS10 | LP | No | No | - |
| 34 | c.1887-5_1904del | 550897 | IVS | IVS13 | P/LP | No | YES, PMID19251731 | Unknown |
| 35 | c.2562-3_2584del | 666179 | IVS | IVS17 | LP | No | No | - |
| 36 | c.153_174+42del | 940843 | IVS | EX1 | LP | Reported in this study, minigene splicing assay | Reported in this study | Reported in this study, fetus |
| 37 | c.2677_2688+7del | 1067321 | IVS | EX18 | LP | No | No | - |
| 38 | c.1136_1563+515del | 962078 | IVS | EX9 | LP | No | No |  |

P, pathogenic; LP, likely pathogenic; Ex, exon; IVS, intervening sequence (intron). Variants locate on the canonical splicing site are in green. Variants locate on the non-canonical splicing site are in blue. Deletion variants affecting splicing site are in read.
